# Single-stage management with combined tri-endoscopic approach for concomitant cholecystolithiasis and choledocholithiasis

**DOI:** 10.1007/s00464-016-4918-6

**Published:** 2016-04-28

**Authors:** Fujing Lv, Shutian Zhang, Ming Ji, Yongjun Wang, Peng Li, Wei Han

**Affiliations:** Department of Gastroenterology, Beijing Friendship Hospital, Capital Medical University, National Clinical Research Center for Digestive Disease, Beijing, 100050 China

**Keywords:** Cholecystolithiasis, Choledocholithiasis, Endoscopic retrograde cholangiopancreatography (ERCP), Laparoscope

## Abstract

**Objective:**

The aim of this study was to investigate the value of a single stage with combined tri-endoscopic (duodenoscopy, laparoscopy and choledochoscopy) approach for patients with concomitant cholecystolithiasis and choledocholithiasis.

**Methods:**

Fifty-three patients with combined gallbladder stones and common bile duct stones from February 2014 to April 2015 were randomized assigned to two groups: 29 patients underwent single-stage surgery with combined duodenoscope, laparoscope and choledochoscope (combined tri-endoscopic group), and 29 patients underwent endoscopic sphincterotomy to remove common bile duct stones followed by laparoscopic cholecystectomy several days later (control group). The success rate of complete stone removal, procedure-related complication, hospital stay and the cost of hospitalization were compared between the two groups.

**Results:**

Altogether, 53 patients (29 patients in combined tri-endoscopic group and 24 patients in control group) successfully underwent the surgery and ERCP procedure. Three patients in the control group developed post-ERCP pancreatitis. One case of bile leaking and one case of residual stone were noted in the combined tri-endoscopic group. There were no significant differences between the two groups with regard to both complete stone removal [96.6 % (28/29) vs. 100 % (24/24)] and procedure-related complication rate [3.4 % (1/29) vs. 12.5 % (3/24)] (*p* > 0.05). No open surgery was required in either group. There were significant differences between the two groups with regard to hospital stay (6.72 ± 1.3 days vs. 10.91 ± 1.6 days, *p* < 0.01) and cost of hospitalization (15,724 ± 1613 CNY vs. 19,829 ± 2433 CNY, *p* < 0.05).

**Conclusion:**

The single-stage combined tri-endoscopic approach for concomitant cholecystolithiasis and choledocholithiasis was just as safe and successful as the control group. In addition, it resulted in a shorter hospital stay and less cost.

With the advent of laparoscopic surgery, the management of concomitant gallbladder stones and common bile duct (CBD) stones has moved from open operation to the combination of laparoscopy and endoscopy. Laparoscopic cholecystectomy (LC) combined with endoscopic spincterotomy (EST) has rapidly become the procedure of choice and is currently widely accepted [1–3]. Clinically, this procedure involves two stages, ERCP and EST followed by LC a few days later or vice versa. This may potentially increase hospital stay and the cost of hospitalization. Here, we compared single stage with combined tri-endoscopic approach using laparoscope, choledochoscope and duodenoscope to the standard two-stage approach in terms of success, complications, hospital stay and cost of hospitalization. This research aimed to develop a more effective management for concomitant cholecystolithiasis and choledocholithiasis.

## Patients and methods

From February 2014 to April 2015, 58 patients with concomitant gallbladder and CBD stones confirmed on ultrasound, abdominal CT scan and MRCP examination were admitted to our center. This study is a 1:1 randomized controlled trial, which was originally designed to select 29 patients for either single-stage combined tri-endoscopic group using laparoscope, choledochoscope and duodenoscope (combined tri-endoscopic group) or control group who underwent ERCP + EST with placement of ENBD tube followed by LC (control group) using random number table. Unfortunately, five patients in control group rejected laparoscopic cholecystectomy after ERCP for stone removal. These five patients were excluded from the study as PPS (per protocol set) was adopted in the data analysis, which can only be restricted to the participants who fulfill the protocol in terms of the eligibility, interventions, and outcome assessment. Therefore, there were 29 patients in combined tri-endoscopic group and only 24 patients in control group.

Exclusion criteria were as follows: intrahepatic biliary stones; a common bile duct stone with the diameter larger than 1.5 cm; patients older than 80 years old; severe cardiopulmonary disease; and contraindications for anesthesia and surgery. The benefits and risks of the procedures were discussed with patients. Informed consents to both the procedure and the study were obtained from all patients.

The Ethics Committee of Beijing Friendship Hospital, Capital Medical University, approved the study protocol (BJFH-EC/2014-047).

## Surgical/endoscopic technique

### Combined tri-endoscopic group

**Fig. 1:**
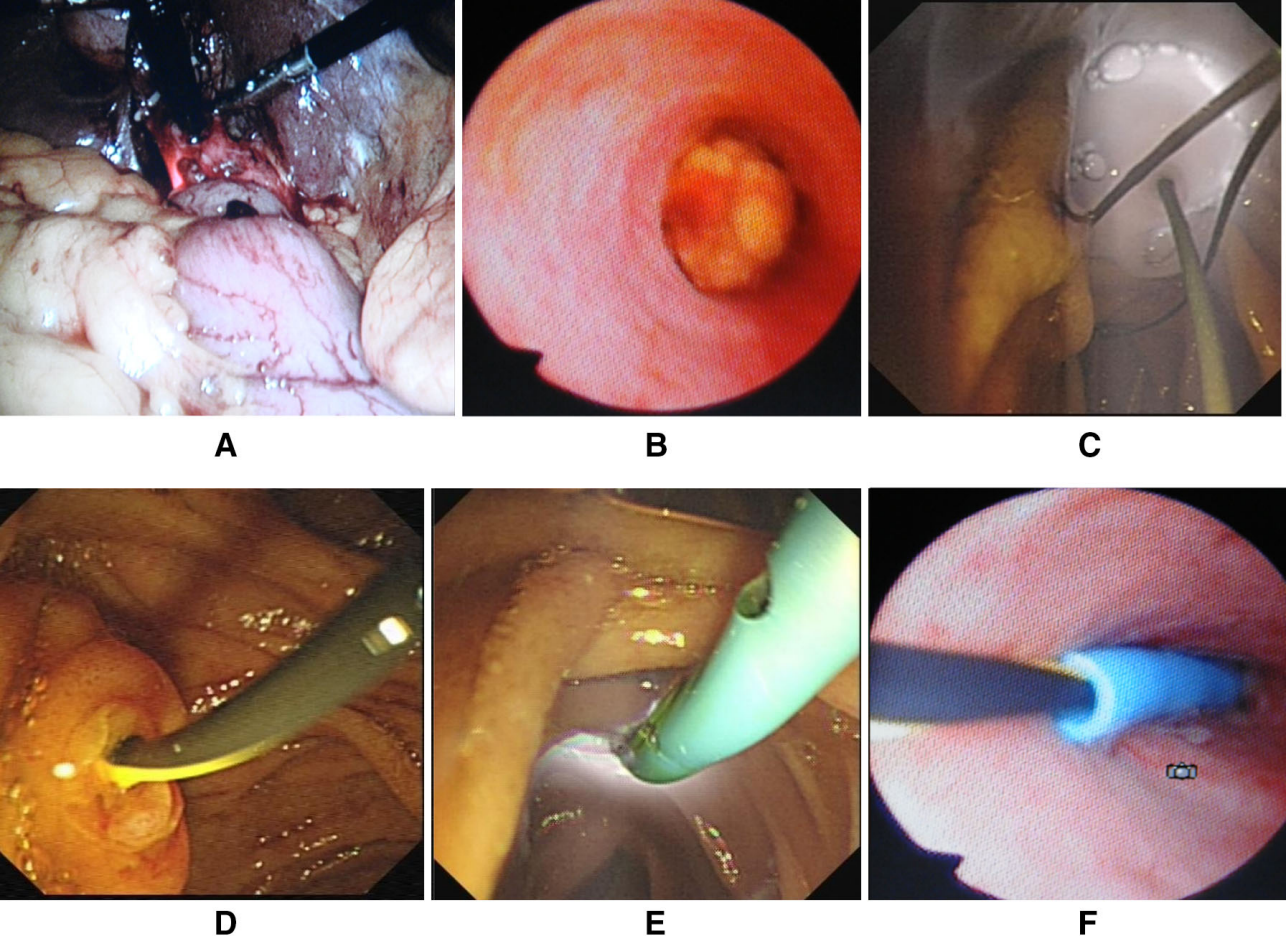
Endoscopy view of various steps of performing single-stage procedure with combined tri-endoscopic approach for patients with concomitant gallbladder stones and CBD stones. **A** Choledochoscope is inserted directly into the dilated cystic duct under laparoscopic view. **B** Choledochoscopic view of common bile duct stone. **C** The guidewire is inserted into the CBD via choledochoscope and arrived at the lumen of the descending duodenum. **D** The hydrophilic end of the guidewire is caught and pulled out of the duodenoscope. **E** An ENBD tube is inserted along the guidewire. **F** The position of the ENBD tube is confirmed by choledochoscope

Under general anesthesia, laparoscopic cholecystectomy was performed. The distal cystic duct was ligated, and then, the gallbladder was removed. Two different procedures were used to remove the CBD stones according to the size of the common bile duct stones and the width of the cystic duct. For the patients with wider cystic ducts and CBD stones less than 1 cm, direct choledochoscopic stone removal was selected. Choledochoscope was inserted directly into the dilated cystic duct under laparoscope, and CBD stones were removed by a basket under direct vision (Fig. 1A, B). For cases with larger CBD stones or narrow cystic ducts, choledochotomy was undertaken, and then, stones were removed by baskets following choledochoscopic exploration. After it was confirmed by choledochoscopy that there were no residual stones in the CBD, a guidewire was inserted into the CBD via choledochoscope, which further passed the duodenal papilla and arrived at the lumen of the descending duodenum. Duodenoscope was inserted into the duodenum, where the hydrophilic end of the guidewire was caught and pulled out of the duodenoscope (Fig. 1C, D). An endoscopic nasobiliary drainage (ENBD) tube was inserted along the guidewire. The position of the ENBD tube was confirmed by choledochoscope (Fig. 1E, F). For all the patients in this group, ENBD tube was kept in place for drainage and further observation. For the patients who underwent choledocholithotomy, primary closure of CBD was undertaken and no T tube was required.

### Control group

Firstly, the patients underwent ERCP and EST for stone removal at the Endoscopy Center. The duodenoscope was inserted into the descending duodenum. After cannulation of the bile duct was achieved, a guidewire was placed. The presence of the CBD stone was confirmed by cholangiography. EST or a combination of EST with CRE balloon dilation was performed. Then, the CBD stones were removed using a basket or balloon. An ENBD tube was inserted and placed.

Then, 2–3 days after ERCP operation, cholangiography was performed via the ENBD tube to demonstrate the presence of residual stones. LC was applied to the patients without residual CBD stones. Patients with residual stones received LC combined with choledochoscopic stone removal trans-cystic duct or choledocholithotomy. ENBD tube was continued after LC. Surgery was postponed for the patients who developed complications after ERCP.

## Postoperative management

Patients in both groups were closely observed for symptoms, such as abdominal pain, fever and melena. Intravenous fluids and prophylactic antibiotics were administered in both groups after LC. Abdominal signs, complete blood count and serum amylase were monitored. Postoperative complications were managed accordingly. Three days postoperation, cholangiography was performed via ENBD tube and the ENBD tube was removed if no residual CBD stones were seen. Elective ERCP was performed for residual CBD stones.

## Outcome measures

### Success rate of stone removal

Success was defined as no residual stones of CBD on ENBD tube cholangiography after surgery. The patients with residual stones after ERCP in the control group were not included.

### Complications

Postoperative complications in the two groups, including pancreatitis, cholangitis, abdominal infection, perforation, bleeding and bile leakage were recorded. The diagnostic criteria for post-ERCP pancreatitis (PEP) include serum amylase three times or more the upper limit of normal, measured 2 h after the procedure plus abdominal pain, fever, nausea, vomiting or peritoneal sign [4]. We adopted Ranson criteria [5] for severe acute pancreatitis. Acute pancreatitis was managed with NPO, NG tube, continuous intravenous somatostatin and antibiotics.

### Hospital stay and cost

Hospital stay was recorded and the cost of hospitalization was calculated for both groups.

### Statistical analysis

Quantitative data were presented as the mean ± SD and tested using a t test. Categorical variables were tested using a Chi-square test. Statistical significance was assumed when *p* < 0.05.

## Results

Fifty-three patients were enrolled in this trial. Twenty-nine were assigned to combined tri-endoscopic group and 24 to control group. Both groups were similar in age, gender and size of CBD stones (Table 1).

**Table 1:** Patient data

	Gender		Age (years)	CBD stone with maximum diameter (cm)
	Male	Female		
Combined tri-endoscopic group	20	9	61.3 ± 14.5	0.9 ± 0.43
Control group	15	9	63.5 ± 12.4	1.0 ± 0.33
*p*	0.62		0.58	0.88

### Success rate

Successful completion of surgery was achieved in all 53 patients. No patients underwent conversion to an open procedure. Although post-ERCP showed small residual stones in two patients in the control group, the stones were retrieved in the following LC. Therefore, postoperative cholangiography demonstrated no residual stones in the control group. In the combined tri-endoscopic group, residual stones were present in one patient. The patient underwent elective ERCP stone removal. There was no significant difference in the success rate of CBD stones clearance for the two groups [96.6 % (28/29) vs. 100 % (24/24); *p* = 0.37]. Among the 29 patients in the combined tri-endoscopic group, 23 patients underwent stone removal via cystic duct (79.3 %) and six had a choledochotomy (20.7 %). In the control group, 22 patients underwent simple LC and two patients underwent further stone removal via cystic duct. No patient in the control group underwent choledochotomy.

### Postoperative complications

Bile leakage was observed in one patient in the combined tri-endoscopic group with daily drainage of 20–80 ml. Bile leaking was resolved via JP drain after 3 days. Three patients in the control group developed postoperative pancreatitis and recovered after NPO, acid inhibition, antibiotics and somatostatin management. There was no cholangitis, abdominal infection, bleeding or perforation. The complication rates showed no significant statistical difference [3.4 % (1/29) vs. 12.5 % (3/24); *p* = 0.21].

### Hospital stay and cost of hospitalization

The average hospital stay of the patients in combined tri-endoscopic group was significantly shorter (6.72 ± 1.3 days) than that of the patients in control group (10.91 ± 1.6 days) (*p* < 0.01). The average cost for combined tri-endoscopic group was significantly lesser than that of control group [15,724 ± 1613 CNY vs. 19,829 ± 2433 CNY; (*p* = 0.03)].

## Discussion

Cholecystolithiasis concomitant with choledocholithiasis is very common, which accounts for 6–20 % of cholelithiasis [1]. The optimal timing and best method for removal of CBD stones associated with gallbladder stones is still controversial. LC is the standard treatment of choice for cholecystolithiasis [3]. However, if it is combined with CBD stones, the conventional management plan is open cholecystectomy, choledocholithotomy and drainage with a T tube. This procedure is more invasive with increased risk of complications. Although trans-cystic duct direct choledochoscopic stone removal under laparoscope can be performed if the cystic duct is large enough or can be dilated, most surgeons become uncomfortable dilating the cystic duct beyond 8 mm in diameter. Stones larger than this are more appropriately dealt with lithotripsy or choledochotomy [6, 7]. ERCP is still a standard procedure to treat CBD stones [1, 4]. However, if it is combined with gallbladder stones, the gallbladder left in place may have a significant risk of stone migrating down to CBD again or an attack of cholecystitis. Costamagna et al. [8] followed 334 CBD stones patients who underwent ERCP for stone removal. They demonstrated the risk of recurrent CBD stones and cholecystitis to be 11.1 and 5.8 %, respectively. Similar results also were reported [9, 10].

With the advent of minimally invasive procedures, the combination of laparoscopy, duodenoscopy and choledochoscopy is proven to be an effective approach for gallbladder stones concomitant with CBD stones. Currently, the majority of cases of cholecystolithiasis with choledocholithiasis have been treated via a two-step management using combination of laparoscope, duodenoscope and choledochoscope, that is, either preoperative ERCP/EST followed by elective LC or LC followed by elective ERCP/EST [11, 12]. Recently, modified tri-endoscopic management has been raised. The procedure includes placing an ENBD tube under duodenoscope, followed by LC and stone removal via choledochoscopy. Postoperative ENBD tube other than T tube is kept. The modified approach has the advantages of high success rate of stone retrieval, gallbladder removal and preservation of the function of Oddi’s sphincter [13]. It also avoids the complications related to T tube placement, such as displacement of the T tube, bile leakage, infection and difficult removal. However, all the treatment plans mentioned above involve two-stage management with ERCP and LC, which causes some discomfort to the patients, an extended hospital stay and increased cost of hospitalization. A single-step procedure combining laparoscopic cholecystectomy, intraoperative ERCP and EST was also investigated [14, 15], but intraoperative cholangiography may not be feasible in some centers.

Our study compared two different approaches of single-stage combining laparoscopy, duodenoscopy and choledochoscopy and two-stage with ERCP + EST followed by LC and identified no significant difference in the success rate of stone removal and treatment-related complications between the two approaches. The single-stage approach had more advantages in terms of shorter hospital stay and lower cost of hospitalization. Unlike ERCP, a retrograde procedure which increases the incidence of pancreatitis, the guidewire insertion in the single-stage approach was an antegrade procedure which has less tissue injury to duodenal papilla. The wire traveled to common bile duct via choledochoscopy, passed the ampulla of vater and arrived at the lumen of the descending duodenum. The guidewire was directed with ease under duodenoscope or even gastroscope. Then, an ENBD tube was inserted under the guidance of the guidewire to the CBD. This method not only avoided the risk of post-ERCP pancreatitis but also preserved the function of the sphincter of Oddi. During the procedure, the lights of the laparoscopy and choledochoscopy should be dimmed to provide an optimal visual field for duodenoscopy, the cooperation and coordination between the gastroenterologists and general surgeons before and during the operation should be critical, and this procedure may not be feasible in some facilities.

In the control group, two-stage operations were performed, in the operating room and endoscopic center, respectively, and three patients developed post-ERCP pancreatitis. The hospital stay was significantly longer than that of the single-stage group. With regard to other complications, the replacement of T tube by ENBD tube allowed the primary closure of the CBD, which in turn decreased the incidence of bile leakage. Mild bile leaking was observed only in one patient in the single-stage group and resolved after conservative treatment. After ERCP, two patients in the control group demonstrated small stones in CBD on cholangiography, which was likely due to the migration of gallstones from gallbladder to extrahepatic bile duct. Therefore, trans-cystic exploration of CBD to examine the residual stones is usually required during LC in two-stage approach.

In our research, the diameters of the CBD stones in our research were less than 1.5 cm, which made the stone removal technically easier; 79.3 % (23/29) of patients succeeded in trans-cystic stone removal in the single-stage group. Further research will focus on trials with larger-sized sample and wider inclusion criteria as well as comparing single-stage approach to open surgery and LC followed by ERCP. With the improvement of facilities, trials on the effectiveness of stone removal via EST during LC will be considered.

In summary, the complementary advantages of the three endoscopes, duodenoscope, laparoscope and choledochoscope, in single-stage approach render an effective, feasible and safe procedure with fewer complications for concomitant gallbladder stones and CBD stones. Single-stage approach reduces the number of procedures, decreases the discomfort to the patients, shortens hospital stay and lowers the cost of hospitalization.
